# Sleep Difficulties in Infancy Are Associated with Symptoms of Inattention and Hyperactivity at the Age of 5 Years: A Longitudinal Study

**DOI:** 10.1097/DBP.0000000000000684

**Published:** 2019-06-03

**Authors:** Hanna Huhdanpää, Isabel Morales-Muñoz, Eeva T. Aronen, Pirjo Pölkki, Outi Saarenpää-Heikkilä, Tiina Paunio, Anneli Kylliäinen, E. Juulia Paavonen

**Affiliations:** * Department of Health, National Institute for Health and Welfare, Helsinki, Finland;; † Pediatric Research Center, Laboratory of Developmental Psychopathology and Children's Hospital, Child Psychiatry, University of Helsinki and Helsinki University Hospital, Helsinki, Finland;; ‡ Institute for Mental Health, School of Psychology, University of Birmingham, Birmingham, United Kingdom;; § Department of Social Sciences, University of Eastern Finland, Kuopio, Finland;; ‖ Department of Pediatrics, Tampere University Hospital, Tampere Center for Child Health Research, Tampere University and Tampere University Hospital, Tampere, Finland;; ¶ Psychiatry, University of Helsinki and Helsinki University Hospital, Helsinki, Finland;; ** Psychology, Faculty of Social Sciences and Humanities, Tampere University, Tampere, Finland.; †† Pediatric Research Center, Child Psychiatry, University of Helsinki and Helsinki University Hospital, Helsinki, Finland

**Keywords:** infant sleep, inattention, hyperactivity, longitudinal study

## Abstract

**Objective:**

Sleep difficulties are associated with cognitive and behavioral problems in childhood. However, it is still unclear whether early sleep difficulties are related to later development. We studied whether parent-reported sleep duration, night awakenings, and parent-reported sleep problems in early childhood are associated with symptoms of inattention and hyperactivity at the age of 5 years.

**Method:**

Our study is based on the Child-Sleep birth cohort initially comprising 1673 families, of which 713 were retained at the age of 5 years. We used the Brief Infant Sleep Questionnaire and the Infant Sleep Questionnaire, which were filled out by the parents when their child was 3, 8, and 24 months and 5 years old. Symptoms of inattention and hyperactivity at the age of 5 years were assessed using the Strengths and Difficulties Questionnaire and the Five-to-Fifteen questionnaire.

**Results:**

Sleep duration at the age of 3, 8, and 24 months was associated with inattentiveness at 5 years of age. Moreover, parent-reported sleep problems at the age of 24 months were related to both inattentive and hyperactive symptoms at the age of 5 years. Finally, at the age of 5 years, parent-reported sleep problems and night awakenings were associated with concurrent symptoms of inattention and hyperactivity.

**Conclusion:**

Our findings suggest that certain sleep characteristics related to sleep quality and quantity in early childhood are associated with inattentiveness and hyperactivity at the age of 5 years. Interestingly, sleep duration in early childhood is consistently related to inattention at the age of 5 years.

A quality sleep is considered essential for children's well-being and development. During infancy and early childhood, sleep has special importance on the brain maturational process, facilitating the development of behavioral and emotional control and higher-order cognition.^1^ However, parent-reported sleep difficulties are very common during infancy/early childhood and associated with inattention/hyperactivity in cross-sectional samples.^2^ Previous studies in healthy preschool-aged and school-aged children indicate that sleep duration is related to inattention^3,4^ and/or hyperactivity^5^ together with other psychiatric symptoms, such as internalizing and externalizing problems.^4^ The associations between night awakenings and inattention/hyperactivity or behavioral problems in typically developing preschoolers are less studied, and the results are partly divergent.^2,6^

Attention-deficit and hyperactivity disorder (ADHD) is one of the most prevalent neurodevelopmental disorders in childhood,^7^ which is characterized by the core psychopathologies of attention difficulties, impulsivity, and hyperactivity.^8^ According to previous meta-analysis, sleep difficulties are more common among children with ADHD in comparison with controls,^9^ with prevalence rates ranging from 29% to 85% in children with ADHD.^10^ There is growing interest in the complex relationship between sleep problems and ADHD symptoms^11,12^ because it has been noticed that sleep deprivation in healthy children can increase ADHD-like symptoms. Accordingly, it has been suggested that early sleep difficulties might impair the development of attentional control.^11^ However, because ADHD does have a hereditary component, parental ADHD and/or other psychiatric risk factors may also negatively affect sleep during infancy.^13,14^

Previous longitudinal studies have reported that sleep difficulties in early childhood predict later cognitive, psychosocial, and physical problems.^15–23^ A few longitudinal studies have also examined the associations between sleep difficulties in childhood and inattentive and/or hyperactive symptoms at the age of 5 to 7 years. Six of these longitudinal studies reported that early sleep difficulties, sleep duration, and/or frequent night awakenings reported by parents are associated with later occurring symptoms of inattention and/or hyperactivity.^15,17–21^ One study did not find any direct relationship between early sleep difficulties and later attentional problems, although sleep difficulties were related to emotional dysregulation, which in turn contributed to poorer attentional functioning.^16^

However, some of these previous longitudinal studies have examined sleep quality using a small number of time points (i.e., 2 or less) and/or very limited number of items (e.g., some studies reporting only 1 sleep item). Moreover, only 1 of these studies covered early childhood, but the earliest measurement time point of sleep difficulties has been at the age of 6 months.^15^ The significance of sleep in infancy needs to be studied because the results of sleep studies in toddlers and preschoolers cannot necessarily be generalized to infants.

Therefore, the aim of this study was to identify whether parent-reported sleep duration, night awakenings, and parent-reported sleep problems during the first years of the child's life (i.e., at the age of 3, 8, and 24 months and 5 years) are associated with symptoms of inattention and/or hyperactivity at the age of 5 years. Based on previous studies,^15,17–21^ we hypothesized that sleep duration, high number of night awakenings, parent-reported sleep problems, or their persistence during early childhood would increase the symptoms of inattention and/or hyperactivity at the age of 5 years.

## METHOD

### Participants

This study is based on a longitudinal child-sleep birth cohort from the Pirkanmaa area, Finland, with several measurement points.^24^ The recruitment and baseline measurement occurred prenatally at the 32nd week, and the follow-up measurements took place at the birth of the child and at the age of 3, 8, 18, and 24 months and 5 years. The families were recruited from health centers in the target area during their normal follow-up visits to the maternity clinics. Data were collected between April 2011 and December 2012, and the infants were born between April 2011 and February 2013. A total of 2244 parents were approved to receive the prenatal questionnaires during their visits to the maternity clinics, of which 1673 (74.6%) families gave their consent to participate in the study and returned the baseline questionnaires. For this study, we used data from the questionnaires at pregnancy (week 32) and when the children reached the ages of 3, 8, and 24 months and 5 years. Parents estimated the quantity and quality of their child's sleep at the age of 3, 8, 18, and 24 months and 5 years and hyperactive and inattentive symptoms at the age of 5 years.

The response rate at the age of 5 years was 42.6% (N = 713). Furthermore, we excluded cases with severe chronic illnesses or disabilities, such as Down syndrome or Hirschsprung disease (n = 10), and co-twins (n = 14). Thereby, the final sample consisted of 689 children with either sleep items and the Strengths and Difficulties Questionnaire (SDQ) or the Five-to-Fifteen (FTF) questionnaire at 5 years of age. All the questionnaires (sleep items, SDQ, and FTF) at the age of 5 years were available for 628 children. Sleep items and the SDQ were available for 656 children, and the FTF questionnaire was available for 661 children. Of the final sample (n = 689), 669 children had parental responses at the age of 3 months, 670 children at the age of 8 months, and 553 children at the age of 24 months.

The respondents at the age of 5 years (n = 689) differed from the nonresponding parents in some demographic characteristics. For instance, the responding mothers were slightly older than the nonresponding mothers (31.2 years compared with 30.7 years; *p* ≤ 0.001) and had higher educational level (*p* ≤ 0.001). However, there were no differences in sex, number of children, birth weight, and maternal smoking during pregnancy.

The study protocol was approved by the local ethical committee (ETL-code R11032). Written informed consent was obtained from all the parents.

### Measures

#### Background Factors

Questionnaires for mothers, both prenatally and at the child's age of 5 years, included several questions on sociodemographic factors and health. The educational level of mothers was defined as primary (elementary school), secondary (high school), or higher (vocational school/university).

#### Child's Sleep Quality and Duration Measures

The Infant Sleep Questionnaire^25^ is a 10-item questionnaire that assesses infant sleeping habits and parental strategies for managing infant sleep. For this study, we used this questionnaire to estimate the average number of parent-reported night awakenings at the age of 3, 8, and 24 months, and parent-reported sleep problems at all time points. Brief night awakenings are quite common in early childhood and a normal part of healthy sleep. In this study, night awakenings were considered those that required resettling. Night awakenings at 3, 8, and 24 months of age were assessed by using the item “How many times does your baby wake each night and need resettling on average.” The categories were 0 = “does not wake,” 1 = “once per night,” 2 = “twice per night,” 3 = “3 times per night,” 4 = “4 times per night,” and 5 = “5 or more times per night.” Furthermore, parent-reported sleep problems were measured using the item “Do you think that your baby has sleep difficulties.” The responses were recoded as a binary variable indicating “no sleeping difficulties” and “mild, moderate, or severe sleeping difficulties.”

Sleep duration was measured using the Brief Infant Sleep Questionnaire.^26^ For this study, we selected items of (1) nocturnal sleep in hours, at all time points; (2) the amount of daytime sleep in hours, at all time points; and (3) the number of awakenings during the night, only at the age of 5 years. At the age of 5 years, parents were asked to evaluate the “average number of night awakenings per night.” A high frequency of night awakenings was defined using the 75th percentile at each time point, based on the sample of this study (i.e., 3, 4, 3, and 1 times per night, respectively).

To examine sleep duration among children at the age of 3, 8, and 24 months and 5 years, total sleep duration was calculated as the sum of daytime and nighttime sleep in hours per day. Extreme outliers were excluded at each time point. More specifically, at the age of 3 and 8 months, we included only those cases with both daytime and nighttime sleep reported. We also excluded cases with total sleep duration less than 7 hours or more than 20 hours because this is out of the normative range and indicates a possible error in the parental estimate. Regarding these outliers, we excluded a total of 14 cases at 3 months and 7 at 8 months. At the age of 24 months, we excluded only those cases (n = 3) with total sleep duration less than 6 hours or more than 18 hours per day. Finally, at the age of 5 years, we excluded only those cases (n = 5) with total sleep duration less than 8 hours or more than 16 hours. Shorter sleep duration per day at each time point was defined using the 25th percentile, based on the sample of this study (13.0, 12.5, 11.4, and 9.8 hours per day, respectively).

**Figure 1.**
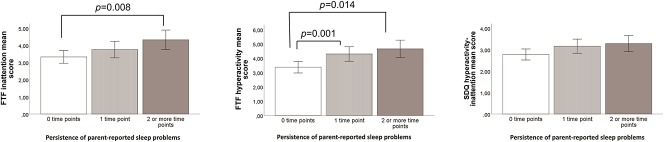
Description of the FTF questionnaire and SDQ mean scores and parent-reported sleep problems persistent over time. Graphs A1–A3 describe the mean scores in inattention and hyperactivity measured using the FTF questionnaire and SDQ, in the persistence of the sleep difficulties, at 0, 1, or 2 or more time points. These graphs show that children with sleep difficulties at 2 or more time points have higher inattention and hyperactivity mean scores measured with the FTF questionnaire compared with children with no sleep difficulties at any time point (A.1-A.2). Only significant results are reported within the graphs. Error bars represent 95% confidence interval. FTF, Five-to-Fifteen; SDQ, Strengths and Difficulties Questionnaire.

To examine the significance of persistent sleep difficulties (i.e., shorter sleep duration, high frequency of night awakenings, and parent-reported sleep problems), new variables were created including the following categories: 0 = “no parent-reported sleep difficulties at any time point,” 1 = “parent-reported sleep difficulties at 1 time point,” and 2 = “parent-reported sleep difficulties at 2 or more time points” (Fig. 1).

#### Hyperactive and Inattentive Symptoms at the Age of 5 Years

Hyperactivity and inattentive symptoms were assessed using 2 different parent-reported questionnaires: the FTF^27^ questionnaire and the SDQ.^28^

The FTF questionnaire comprises 181 statements with 3 response alternatives for 5- to 15-year-olds, related to behavioral or developmental problems. In this study, we used 18 items reflecting the same symptoms as found in the DSM-IV criteria for attention-deficit and hyperactivity disorder (ADHD), comprising the 9-item inattention domain and the 9-item hyperactivity-impulsivity domain. The FTF inattention total score was the sum of 9 inattention items, and the FTF hyperactivity-impulsivity total score was the sum of 9 hyperactivity-impulsivity items. The cutoff point for both FTF inattention and hyperactivity-impulsivity domain was 6 points or more, corresponding to the 75th percentile in our 5-year-old sample.

The SDQ is a brief behavioral screening questionnaire for 3- to 16-year-olds including 25 questions. Parents rate the statement best describing their child's behavior on a 3-point scale. In this study, we used only the 5-item hyperactivity-inattention scale. The total scale score was the sum of 3 items and 2 reversed items. The cutoff point for hyperactive/inattentive symptoms was 5 points or more, corresponding to the 75th percentile of the 5-year-old children in this study.

#### Covariates

For covariates, we selected those variables that were also considered in previous longitudinal studies^15–21^ and those that were significantly associated with any of the sleep and ADHD variables in our sample. The final partially adjusted model controlled for the child's age at 5 years, sex, the mother's educational level, and whether the mother smoked during pregnancy. The final fully adjusted model additionally controlled for birth weight, the mother's age, and the number of children in the family during pregnancy.

### Statistical Analysis

Statistical analyses were performed using SPSS Statistics V25.0. First, the distribution of the variables of interest was described. Second, to compare the prevalence of elevated inattention and hyperactivity symptom scores (scoring over 75th percentile in the inattentive or hyperactive scale score) between children having sleep difficulties (shorter sleep duration, higher frequency of night awakenings, parent-reported sleep problems) versus children without sleep difficulties, χ^2^ tests were used.

Third, to examine the potential effects of sleep difficulties at the age of 3, 8, and 24 months (i.e., predictors) on the child's hyperactive and/or inattentive symptoms at the age of 5 years (i.e., outcomes), a series of multivariate linear regression analyses were conducted. Among the independent variables, parent-reported sleep problems and night awakenings were treated as dichotomous variables (no vs yes), whereas sleep duration and hyperactive and/or inattentive scale scores were used as continuous variables. Each explanatory variable of interest, along with covariates, was studied in different models.

Fourth, the means of inattentive and hyperactive scale scores of the FTF questionnaire and SDQ in children with no parent-reported sleep difficulties (shorter sleep duration, higher frequency of night awakenings, parent-reported sleep problems) at any time point, children with parent-reported sleep difficulties at 1 time point, and children with parent-reported sleep difficulties at 2 or more time points were compared using analysis of variance.

Finally, additional multivariate logistic regression analyses were conducted to examine how shorter sleep duration at different time points (at the age of 3, 8, and 24 months and 5 years), adjusted by the covariates, predicted over 75th percentile cutoff scores in hyperactive and/or inattentive scales of the FTF questionnaire and SDQ.

## RESULTS

Background factors of the children and their families are reported in Table 1. Table 2 shows the prevalence of some sleep difficulties (shorter sleep duration, high frequency of night awakenings, and parent-reported sleep problems) at different time points in those children having hyperactivity-impulsivity and/or inattentive scale scores (i.e., >75th percentile) at the age of 5 years.

**Table 1.**
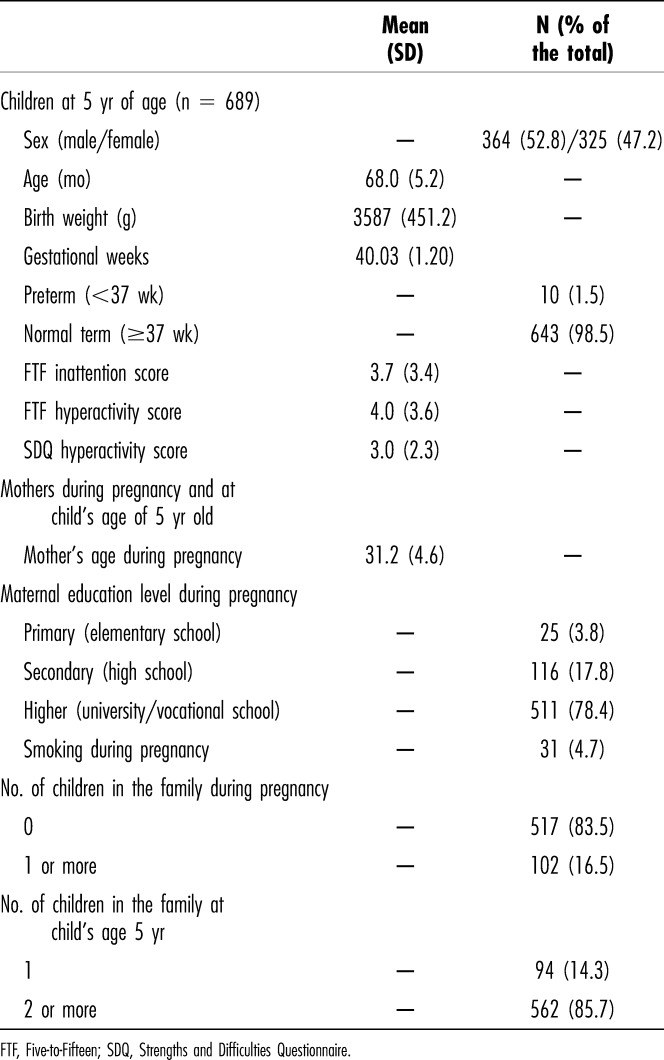
Descriptive Variables of Children at the Age of 5 Years and Mothers During Pregnancy

**Table 2.**
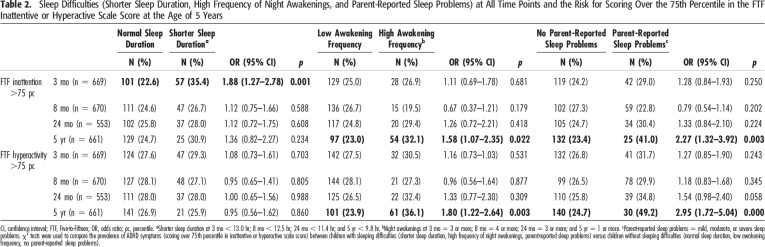
Sleep Difficulties (Shorter Sleep Duration, High Frequency of Night Awakenings, and Parent-Reported Sleep Problems) at All Time Points and the Risk for Scoring Over the 75th Percentile in the FTF Inattentive or Hyperactive Scale Score at the Age of 5 Years

Our main results reported in Table 3 showed, in both partially and fully adjusted models, that sleep duration at the age of 3 and 8 months was significantly associated with inattentive symptoms measured with the Five-to-Fifteen (FTF) questionnaire at the age of 5 years. The fully adjusted model also showed a significant association between sleep duration at 24 months and inattentive symptoms at 5 years. Moreover, parent-reported sleep problems at the age of 24 months and 5 years were related to inattentive and hyperactive-impulsive symptoms measured with the FTF questionnaire at the age of 5 years. Finally, a tendency for night awakening at the age of 5 years was related to concurrent inattentiveness and hyperactivity-impulsivity. All these results remained when the parents with a prior attention-deficit and hyperactivity disorder diagnosis were excluded.

**Table 3.**
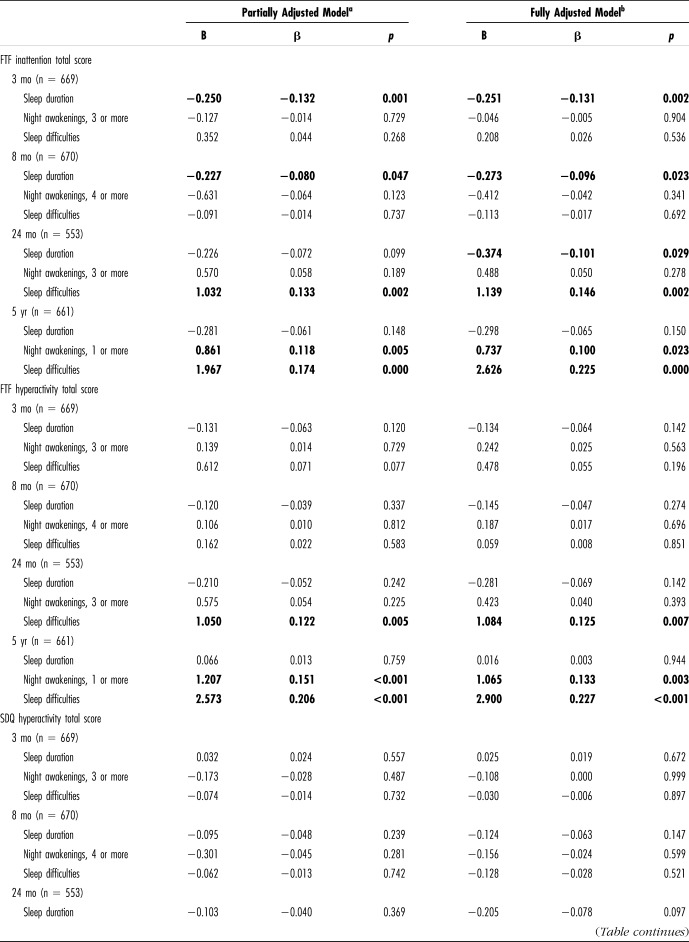

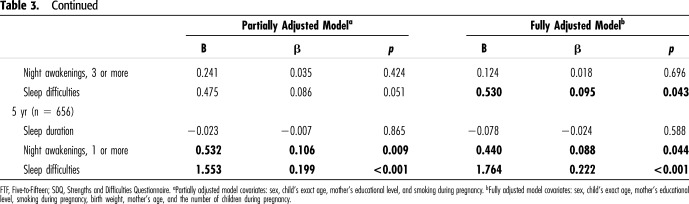
Multivariate Linear Regression Models Between Sleep Difficulties at the Age of 3, 8, 24 Months and 5 Years and Inattention and Hyperactivity Total Scores at the Age of 5 Years

To study risks related to shorter sleep duration, we found on the basis of logistic regression models that shorter sleep duration (<13.0 hours per night) at the age of 3 months predicted inattentiveness (FTF inattentive scale score over the 75th percentile) at the age of 5 years [partially adjusted odds ratio (OR) = 1.97, 95% confidence interval (CI) = 1.31–2.95, *p* < 0.01; and fully adjusted OR 1.93, 95% CI = 1.24–3.01, *p* < 0.01, respectively]. However, regarding the other time points, no significant associations were found between shorter sleep duration and inattentive and/or hyperactive-impulsive symptoms.

Finally, children with persistent parent-reported sleep problems (i.e., prevalence of parent-reported sleep problems at 2 or more time points) had higher inattentive (*p* < 0.01) and hyperactive-impulsive (*p* < 0.01) scale scores measured with the FTF questionnaire (Fig. 1). Furthermore, children with shorter sleep duration at 2 or more time points had higher inattentive (F = 4.4, *p* < 0.05) scale scores measured with the FTF questionnaire compared with other children.

## DISCUSSION

Our main findings suggest that sleep duration at the age of 3, 8, and 24 months was associated with inattentiveness in 5-year-old children. Night awakenings (≥1 per night) were associated with inattentiveness and hyperactivity-impulsivity at the age of 5 years. The parent-reported sleep problems in their 24-month-old and 5-year-old children were related to symptoms of inattentiveness and hyperactivity-impulsivity.

This is the first longitudinal study evaluating the link between shorter sleep duration already from 3 months of age and later inattentive symptoms. Although there are a number of cross-sectional studies showing an association between symptoms of inattentiveness and hyperactivity-impulsivity and sleep problems, longitudinal studies are few. The existing studies show that various sleeping problems in children predict symptoms of inattentiveness and hyperactivity-impulsivity; however, because sleep in infants is very different than sleep in toddlers and older children, the findings cannot directly be extrapolated to concern infants.

After our initial hypothesis, we found consistent associations between sleep duration at the age of 3, 8, and 24 months and inattentive symptoms at the age of 5 years. These results are in line with previous cross-sectional studies conducted in healthy preschool/school-aged children and adolescents. These studies have reported an association between sleep duration and inattentive symptoms rated by a parent or teacher or shown in cognitive tests.^3,4^ In addition, subjectively or objectively measured sleep duration at the age of 12 to 18 months has also been linked with later attentional problems at the age of 3 to 5 years.^21,23^

It is a novel and interesting finding that sleep duration in early childhood was consistently related to later signs of inattentiveness. Reportedly, children with attention-deficit and hyperactivity disorder (ADHD) have reduced sleep quantity.^12^ Thus, a shared genetic/neurobiological background involving the functioning of the frontal brain areas may account for the relationship between early shorter sleep duration and later occurring inattentive symptoms. On the other hand, it might also be possible that severe sleep difficulties in early childhood could impair the development of self-regulatory systems. The development of attentional control may be particularly vulnerable to the effects of sleep deprivation and/or sleep problems during the infancy and preschool period because of its prolonged course of maturation.^1^ There is evidence that experimental sleep deprivation in adults leads to reduced brain activation in multiple brain areas, including the bilateral parietal lobule and prefrontal cortex.^29^ Clearly, further studies are needed to explore the biological mechanisms beyond the reported association, which is now quite consistently reported in several longitudinal studies in later stages. If these mechanisms are confirmed, the findings would be clinically highly significant, underlining the importance of the recognition and treatment of sleep difficulties in early childhood.^11^

In the present study, we did not find any longitudinal associations between sleep duration, night awakenings, parent-reported sleep problems during infancy, and later hyperactivity-impulsivity. So far, longitudinal studies analyzing sleep duration and hyperactivity/impulsivity are partly contradictory. For instance, relatively strong associations between subjectively and/or objectively measured shorter nighttime sleep duration and parent-reported hyperactivity/impulsivity have been reported in cross-sectional studies among healthy preschool-aged and school-aged children.^5^ Furthermore, and in contradiction to our findings, 2 longitudinal studies have reported the link between parent-reported shorter nighttime sleep duration before the age of 3.5 years and symptoms of hyperactivity,^18,19^ but not inattentiveness, at the age of 5 to 6 years.^18^ However, there is also some evidence in later childhood that supports that children diagnosed with ADHD at a later stage slept significantly less than their peers from the age 5 to 9 years, and the main reason for the shorter sleep duration was later bedtimes.^15^

Our hypothesis concerning the association between frequent night awakenings during the infancy/preschool period and later occurring inattentive and/or hyperactive-impulsive symptoms was only partially supported because we only found an association between frequent night awakenings and inattentive/hyperactive symptoms at the age of 5 years. These results were similar to a previous longitudinal study, in which children with a later diagnosis of ADHD had significantly more night awakenings from the age of 5 years.^15^ However, and contrary to our findings, frequent night awakenings at the age of 18 months have been found to be related to attentional and behavioral problems at the age of 5 to 6 years.^20,21^ Furthermore, 1 cross-sectional study with preschool-aged children showed that night awakenings at the age of 2 to 5 years were only related to hyperactivity in boys.^6^ Night awakenings in infants can be caused by different factors such as breastfeeding, parental involvement when falling asleep, cosleeping with the infant, the child's challenging temperament,^30^ and locomotion.^31^ Therefore, early night awakening probably represents an etiologically heterogeneous group. In addition, it is worth noting that although night awakening may indicate clinically significant problems, some of them are normative.

Finally, our study showed that children with persistent parent-reported sleep problems/shorter sleep duration showed higher inattentive and/or hyperactive-impulsive scale scores compared with other children, which is consistent with a previous longitudinal study.^17^ However, persistence of frequent night awakenings was not associated with inattentive and hyperactive-impulsive scale scores. Previous research shows that there is a wide natural variability in sleep patterns throughout childhood.^15,23^ Our study suggests that sleep problems at different time points might be significant risk factors for inattentive and/or hyperactive-impulsive symptoms at the age of 5 years.

This study has a number of strengths, including the use of several measurement points that cover infancy, toddlerhood, and preschool ages; the characterization of parent-reported sleep difficulties within 3 different domains (i.e., sleep duration, night awakenings, and parent-reported sleep problems); and the examination of sleep functioning from very early stages (i.e., at 3 months of age). However, there are some limitations that should be also mentioned. First, a rather high prevalence of nonresponding families when their child reached 5 years of age may have affected these results. We observed that mothers who responded when their child was 5 years old were more highly educated and older than nonresponding mothers. A mother's low educational level is associated with higher scores in questionnaires measuring inattention and/or hyperactivity-impulsivity.^32^ This way, the children of our responding mothers may be at lower risk for ADHD symptoms than those in the general population. Therefore, this bias is likely to weaken the findings in this dataset because it may lower the prevalence of children with a risk of ADHD. Second, we used only subjective measurements of sleep and attention issues, which may not be sensitive enough when measuring these symptoms. Furthermore, day-to-day variability in sleep quality cannot be only measured using questionnaire data. Because subjective reports and objective reports may have discrepancies at least in some of the cases, future studies on the topic should aim to confirm these findings using objective sleep measures, such as actigraphy. Third, parenting strategies or lack thereof may be related to both sleep difficulties during infancy and child's later inattentive symptoms.^14^ In addition, many other environmental factors are also likely to affect sleep in early childhood. Finally, we did not include reports from other sources (e.g., daycare teachers) on inattentive and/or hyperactive symptoms in our study.

In conclusion, in the present study, sleep duration at the age of 3, 8, and 24 months was associated with symptoms of inattention at the age of 5 years. Furthermore, sleep difficulties at the age of 24 months were related to inattentive and hyperactive-impulsive symptoms at the age of 5 years. In addition, parent-reported sleep problems and night awakenings at the age of 5 years were associated with these symptoms supporting earlier findings of the associations between present sleep and inattention and hyperactivity.^4^ Our study supports previously reported findings on the association of poorer sleep with shorter duration and more night awakenings in children with symptoms of inattention and/or hyperactivity and suggests that shorter sleep duration can start as early as 3 months of age. Taking into account the high prevalence of sleep problems in healthy samples during the infancy and preschool period,^2,22^ our results emphasize the importance of the early assessment and treatment of sleep difficulties because they may increase the risk for future developmental difficulties. Our current study adds significance to previous studies by showing that the altered developmental pathway of sleep quality and quantity occurs already at an early stage of infancy among those children who might later have attention problems. Recognizing children at risk for ADHD as early as possible might provide an opportunity to support these families already before the diagnosis of ADHD is made. Although a diagnosis of ADHD is often given much later than at the age of 5 years, symptoms of inattention and hyperactivity at the age of 5 years increase risk for ADHD diagnosis later in life.^33^ In future, register-based studies on children with early sleep difficulties and later diagnosis of ADHD would be of interest to find out whether early sleeping difficulties also predict a diagnosis of ADHD.
